# Limited Service Availability, Readiness, and Use of Facility-Based Delivery Care in Haiti: A Study Linking Health Facility Data and Population Data

**DOI:** 10.9745/GHSP-D-16-00311

**Published:** 2017-06-27

**Authors:** Wenjuan Wang, Michelle Winner, Clara R Burgert-Brucker

**Affiliations:** a The Demographic and Health Surveys (DHS) Program, ICF, Rockville, MD, USA.

## Abstract

**Background:**

Understanding the barriers that women in Haiti face to giving birth at a health facility is important for improving coverage of facility delivery and reducing persistently high maternal mortality. We linked health facility survey data and population survey data to assess the role of the obstetric service environment in affecting women's use of facility delivery care.

**Methods:**

Data came from the 2012 Haiti Demographic and Health Survey (DHS) and the 2013 Haiti Service Provision Assessment (SPA) survey. DHS clusters and SPA facilities were linked with their geographic coordinate information. The final analysis sample from the DHS comprised 4,921 women who had a live birth in the 5 years preceding the survey. Service availability was measured with the number of facilities providing delivery services within a specified distance from the cluster (within 5 kilometers for urban areas and 10 kilometers for rural areas). We measured facility readiness to provide obstetric care using 37 indicators defined by the World Health Organization. Random-intercept logistic regressions were used to model the variation in individual use of facility-based delivery care and cluster-level service availability and readiness, adjusting for other factors.

**Results:**

Overall, 39% of women delivered their most recent birth at a health facility and 61% delivered at home, with disparities by residence (about 60% delivered at a health facility in urban areas vs. 24% in rural areas). About one-fifth (18%) of women in rural areas and one-tenth (12%) of women in nonmetropolitan urban areas lived in clusters where no facility offered delivery care within the specified distances, while nearly all women (99%) in the metropolitan area lived in clusters that had at least 2 such facilities. Urban clusters had better service readiness compared with rural clusters, with a wide range of variation in both areas. Regression models indicated that in both rural and nonmetropolitan urban areas availability of delivery services was significantly associated with women's greater likelihood of using facility-based delivery care after controlling for other covariates, while facilities' readiness to provide delivery services was also important in nonmetropolitan urban areas.

**Conclusion:**

Increasing physical access to delivery care should become a high priority in rural Haiti. In urban areas, where delivery services are more available than in rural areas, improving quality of care at facilities could potentially lead to increased coverage of facility delivery.

## Abstract (precis)

Proximity to a health facility offering delivery services and readiness of the facilities to provide such services were poor in both rural and urban areas outside of Port-au-Prince. Availability of a proximate facility was significantly associated with women in rural and urban areas delivering at a facility, as was the quality of delivery care available at the facilities but only in urban areas.

## INTRODUCTION

Haiti has the highest maternal mortality ratio in Latin America and the Caribbean, at an estimated 359 deaths per 100,000 live births.^1^ Every year, thousands of women in Haiti die from causes that could be prevented by access to comprehensive and skilled obstetric care during pregnancy, childbirth, and the postpartum period.^2^^,^^3^ Use of maternal health services, especially facility delivery, remains low in Haiti. Only 36% of births take place in health facilities, according to the 2012 Haiti Demographic and Health Survey (DHS).^4^ Unless a woman delivers at a health facility, she is unlikely to have access to emergency obstetric care, which is considered the most important strategy for reducing maternal deaths.^5^^,^^6^ Increasing use of facility delivery is critical for reducing maternal mortality in Haiti.

Increasing use of facility delivery is critical for reducing maternal mortality in Haiti.

An extensive body of literature exists on factors that influence facility delivery.^7^^–^^11^ The majority of studies have focused on the demand side—for example, the characteristics of women and their families. A few studies have looked at social contextual factors such as community norms, media access, and the level of local development.^12^^–^^15^ In Haiti, a few studies have found that facility delivery was associated with maternal and birth characteristics, household poverty, use of antenatal care services, and community exposure to mass media.^16^^,^^17^

The effect of the supply side—delivery care offered in health facilities—on facility delivery has received only limited attention.^8^^,^^11^ One of the main reasons that research on the effects of service provision is limited is lack of suitable data. Supply-side data typically come from health facilities and need to be linked to data from population-based surveys in order to explore the relationship between the provision of services and women's use of facility-based delivery care.

With the availability of geographic data from both household surveys and health facility surveys, it has become possible to link population data and health facility data within a geographic information system (GIS). A few studies have linked DHS data and facility census data to assess how distance to the closest facility affects women's use of reproductive health services.^18^^–^^20^ For example, a study in Malawi and Zambia linking DHS clusters and health facilities (from a facility census) found that in both countries a longer straight-line distance from the DHS cluster to the closest facility offering emergency obstetric care significantly reduced the likelihood of facility delivery.^19^ Another study in Zambia with the same methodology but focusing on antenatal care found that distance to the closest facility had a significant effect on the content of care women received but had no effect on number of antenatal care visits or timing of the first visit.^18^ In a rural setting in Ghana, Nesbitt and colleagues linked health facility census data and health and demographic surveillance data from about 600 villages and found a significant association between distance to the closest delivery facility and women's likelihood of delivering in a health facility.^20^

While these studies have contributed to establishing a geospatial methodology to assess the relationship between service provision and use, linking DHS clusters and closest facilities is subject to misclassification errors. Because DHS cluster coordinates are displaced before release of the data (to ensure respondent confidentiality is maintained), the closest facility identified based on the released geographic data may not be the nearest facility in reality. Skiles and colleagues indicated that, due to displacement of cluster coordinates, the distance to the closest facility can be misclassified for 34% to 43% of clusters.^21^ The displacement is an important limitation of the data, so instead of only looking at the closest facility our study measured the service environment (all available facilities) within a reasonable distance of the displaced cluster, thus representing the likely service environment of the real cluster location.

Because of Haiti's mountainous terrain, physical accessibility remains one of the biggest barriers to using health care.^22^^,^^23^ Gage and Guirlène Calixte linked women's reports on use of facility delivery care and community key informants' reports on health services and found that in rural Haiti the physical accessibility of health facilities was strongly associated with women's use of delivery care.^23^ Women's odds of being attended by a skilled birth attendant were positively associated with the presence of a health worker providing antenatal care in the neighborhood but negatively associated with living in a mountainous terrain and with distance from the nearest hospital. Ruktanonchai and colleagues found that in the 5 East Africa countries studied, geographic inaccessibility was an important predictor of use of maternal health care services, including skilled birth attendance.^24^ This furthers the argument that straight-line distance linkage between a facility and a DHS displaced cluster may cause misclassification in the Haiti context. Thus, the service environment approach is a better approximation of the likely access to health facilities for a woman.

Physical accessibility remains one of the biggest barriers to using health care in Haiti due to the mountainous terrain.

While physical access is important, another key determinant of service utilization is the quality of care. Families may bypass the nearest health facility when quality is an issue.^25^^–^^27^ In examining the effect of the quality of care on use of services, some studies have looked at the quality of care from the user's perspective.^15^^,^^28^ While this perspective is indicative, it is subject to the respondents' level of knowledge about the services provided at health facilities, which may be biased or misinformed. Among the limited number of studies based on linked population data and facility data, a few have looked at the quality of service provision in health facilities. The study in Zambia by Kyei and colleagues measured the level of care using an index that combined several process and structural aspects of antenatal care provided at facilities.^18^ The level of service provision at the closest health facility was found to be significantly associated with the content of antenatal care received. In Nepal, when quality of care was measured solely in structural terms (e.g., infrastructure, availability of medicine, number of staff) a significant effect was also seen on the use of antenatal care and immunization services.^29^

Another key determinant of service utilization, beyond physical proximity, is the quality of care of services.

Haiti has a hierarchical system of health care provision in which small facilities are located in villages or small communities and larger, better-equipped facilities are located in cities. Small or low-level facilities may not provide delivery services, however, and among those that provide these services there is little information on how prepared they are to provide good-quality delivery care and how their service preparedness affects utilization. The Haiti 2013 Service Provision Assessment (SPA) and 2012 DHS provide an opportunity to link data for health facilities and DHS clusters in order to explore the influence of service availability and readiness on use of delivery care in facilities.

## DATA AND METHODS

### Data

The Haiti DHS provides data on women's socio-demographic characteristics and their use of maternal health care services, including facility-based delivery care. The Haiti SPA provides information on the availability of delivery care at health facilities and the readiness of facilities to provide good-quality services. This study used geographic data collected in both surveys to link DHS clusters and SPA facilities.

This study used geographic data collected in the Haiti Demographic and Health Survey (DHS) and the Service Provision Assessment (SPA) to link DHS clusters and SPA facilities.

#### Population Data

The 2012 Haiti DHS is a population-based household survey that provides representative estimates for both urban and rural areas and for the 10 administrative departments of Haiti. The survey used a 2-stage cluster sampling design. At the first stage, 445 clusters were selected with probability proportional to their population size from a master national sample frame. At the second stage, a systematic sample of households was drawn in each of the selected clusters. All women ages 15–49 in the sampled households were eligible for individual interview, which collected data on their socio-demographic characteristics and use of health care services. A total number of 14,287 women were interviewed.

The Haiti DHS georeferenced the locations of the sampled clusters by using Global Positioning System (GPS) receivers to collect the coordinates of the center of the populated areas of the clusters. Prior to release of the geographic dataset, the cluster coordinates were verified and geographically displaced.^30^ Coordinates of urban clusters were displaced up to a maximum distance of 2 kilometers (km); average urban displacement was 0.8 km. In rural areas, the displacement distance was up to 5 km with a further, randomly selected 1% of rural clusters displaced up to 10 km; average rural displacement was 2.1 km. These displaced GPS locations were used in our analysis.

Given that the outcome of interest was facility delivery, we limited the analysis to women who had a live birth in the 5 years preceding the survey (N=5,218) with a focus on their most recent birth. We excluded 234 women interviewed in 45 camp clusters that housed the population displaced by the 2010 earthquake because they were likely to have lived in a different area when they had their most recent birth. Thus, the health care environment where they had the birth was likely different from where they had been surveyed. Another 63 women were excluded from the analysis because they were from 8 clusters with missing georeferenced data. The final analysis sample consisted of 4,921 women from 392 clusters with GPS data available.

We limited our analysis to women who had a live birth in the 5 years preceding the survey, with the final analysis sample comprising 4,921 women.

#### Health Facility Data

The 2013 Haiti SPA is a health facility census of 907 public and private facilities, from hospitals at the highest level to dispensaries at the lowest level. The SPA provides data on availability of key health services and readiness to provide these services. Data were collected using 4 types of survey instruments: the inventory questionnaire, health provider interviews, observation of consultations, and client exit interviews. For our analysis, facility data primarily came from the inventory questionnaire, which was administered to the facility manager or the most knowledgeable person for specific service areas. The inventory questionnaire collects data on the facility's infrastructure, supplies, medicines, staffing, trainings, and routine practices in providing general and specific services.

**FIGURE 1:**
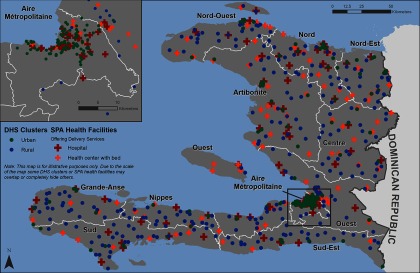
Geographic Distribution of 2012 DHS (Displaced) Clusters and 2013 SPA Facilities Included in the Analysis Abbreviations: DHS, Demographic and Health Survey; SPA, Service Provision Assessment.

The SPA georeferenced the locations of the health facilities by using GPS receivers. Unlike the DHS GPS data for each cluster, facilities' GPS coordinates were not displaced. Data on 195 hospitals and health centers with a bed that offer delivery services were analyzed in this study. We excluded health centers without a bed and dispensaries from the analysis because they are not mandated to provide delivery care and are rarely used for delivery care.^4^^,^^31^ Figure 1 shows the location of the SPA facilities as well as the DHS (displaced) clusters that were included in the analysis.

Data on 195 hospitals and health centers with a bed that offered delivery services were analyzed.

#### Linked Data Between DHS Clusters and SPA Facilities

Using publicly available GPS data for DHS (displaced) clusters and SPA facilities, we linked facilities to clusters to measure the health service environment of the clusters. We first created a distance matrix with the direct distance measurement from every DHS (displaced) cluster location to every health facility. The distances were then used to identify facilities within a 5-km buffer distance from an urban cluster and a 10-km buffer distance from a rural cluster. These buffer sizes around the displaced location were chosen to ensure that facilities within the same distance around the real (non-displaced) location of the cluster were included within the buffer, given the displacement radius used for urban (maximum 2 km) and rural locations (maximum 10 km). We also believe that facilities within the chosen buffer distance reasonably represent the service environment where individuals seek health services, if not the exact facilities they visit. Lastly, data from all the facilities within the buffer distance were summarized for each cluster to measure the cluster's service environment.

##### Definitions of Key Measurements

The outcome variable was dichotomous, indicating whether a woman delivered her most recent birth at a health facility. We measured the service environment with 2 indicators: availability of facilities offering delivery care and facilities' readiness to provide good-quality delivery care. Both were measured at the DHS cluster level and derived from the facility-level data after linking facilities to DHS clusters.

At the facility level, we measured availability using the SPA definition of facilities offering delivery care, which the SPA obtained by asking the facility manager if the facility provides delivery services. We measured facility's readiness to provide good-quality delivery care by a readiness score created with principal component analysis based on a set of service readiness indicators defined by the World Health Organization.^32^ For each indicator—according to whether the facility met the criteria for availability—facilities were assigned a binary variable: 1=available, 0=unavailable. A total of 37 readiness indicators were constructed; their definitions are presented in Supplementary Table 1. The readiness score was computed based on the first component resulting from the principal component analysis, which explained the largest proportion of the total variance. The readiness score is a relative summary indicator of how ready a health facility is to provide good-quality delivery services. A higher score represents better readiness and a lower score represents poorer readiness compared with other facilities. Availability of delivery service and readiness indicators were then summarized to the DHS cluster level, as described below.

We measured a facility's readiness to provide good-quality delivery care by a readiness score based on 37 indicators defined by WHO.

To measure availability of delivery services, we counted the number of facilities offering delivery services within the specified buffer of each DHS cluster. We categorized clusters into groups with 3 levels of availability:

1. Low availability: no facility with delivery services within the buffer distance
2. Medium availability: 1 facility with delivery services within the buffer distance
3. High availability: 2 or more facilities with delivery services within the buffer distance

We categorized clusters into groups with 3 levels of availability of delivery services: low, medium, or high.

Facilities' readiness to provide delivery care was measured by the median readiness score of all the facilities within the buffer. Given that the readiness score is a relative measurement, we divided the clusters into low-, medium-, and high-level readiness groups based on the score terciles at the cluster level. Clusters with a median score (median score of all facilities within the buffer) falling in the top 33% were considered in the high-level readiness group; those with a median score in the bottom 33% were categorized into the low-level readiness group; and the rest were put in the medium-level readiness group.

We measured facilities' readiness to provide delivery services by the median readiness score of all the facilities within the buffer and divided clusters into low-, medium-, or high-level readiness groups based on the score terciles.

### Statistical Analysis

The analysis was stratified by urban and rural residence because of differences between urban and rural areas in the health service environment and women's health care-seeking behaviors. We further separated the Port-au-Prince metropolitan area (comprising the capital city Port-au-Prince and the urban zones of the Ouest region) from other urban areas because of the substantial differences in the density and types of health facilities.

Random-intercept logistic regressions were used for the multivariable analysis. DHS data follow a hierarchical structure—that is, individuals are nested within households and households are nested within clusters. Respondents who live in the same household or cluster may not be independent of one another. Moreover, the outcome variable is at the individual level but the key exploratory variables—level of service availability and readiness of facilities to provide delivery services—are at the cluster level. A multilevel analysis approach is more appropriate to allow for simultaneous investigation of the effect of the group-level and individual-level predictors on individual-level outcomes.^33^ Therefore, we applied multilevel (individual- and cluster-level) random intercept logistic models to investigate how the service environment measures affect women's use of facility-based delivery care. The household level was omitted since there were few women who had a child in the 5 years preceding the survey living in the same household.

Other variables adjusted for included women's age at birth, birth order, mother's education, household wealth quintile, number of antenatal care visits, and region (department), all of which have been found to be associated with facility delivery.^12^^,^^34^

## RESULTS

### Sample Characteristics

For health facilities, we analyzed data on hospitals and health centers with a bed that provide delivery services. The distribution of these facilities in each residence location by facility background characteristics is presented in Supplementary Table 2. Overall, hospitals and health centers each accounted for half the facilities; but in the metropolitan area most facilities offering delivery services were hospitals, while in rural areas most were health centers. With regard to the managing authority, more than half of the facilities in the metropolitan area were private for-profit, while government health facilities were more common in other urban areas and rural areas. Facility distribution also varied across regions in rural and other urban areas. In rural areas, some regions, such as the Ouest region, had many health facilities, while some, such as Sud-Est and Grand-Anse, had only a few.

Most facilities offering delivery services were hospitals in the metropolitan area and health centers in rural areas.

For use of facility-based delivery services, data were based on 4,921 women who had a live birth in the 5 years preceding the DHS survey (including 2,878 women in rural areas, 1,214 in the metropolitan area, and 829 in other urban areas). Supplementary Table 3 provides the distribution of these women by background characteristics and receipt of antenatal care for the most recent birth. In all 3 location categories, a majority of the women reported having their most recent birth between ages 20–34, while 14% to 15% were under age 20 when they had their most recent birth. Most women in the metropolitan and other urban areas had 3 children or fewer, with about 40% having the most recent birth as their first birth. In contrast, in rural areas a higher percentage of women (38%) had 4 children or more. In the metropolitan and other urban areas, most women reported having a primary education and close to 60% had secondary or higher education. In contrast, in rural areas only 25% of women had secondary or higher education, while 27% had no education. In the metropolitan and other urban areas, women in the study were primarily in the upper 3 wealth quintiles, while in rural areas 71% of women were in the poorest 2 quintiles. As for women's receipt of antenatal care during pregnancy for their most recent birth, more than three-quarters of women in the metropolitan and other urban areas had 4 or more antenatal care visits compared with less than two-thirds (60%) of rural women.

### Availability of Delivery Services

**FIGURE 2:**
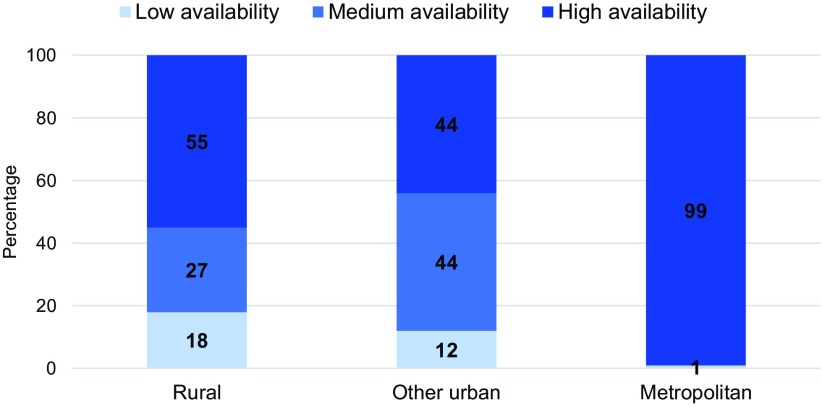
Percent Distribution of Women by Level of Availability of Facilities With Delivery Services Within the Buffer Distance,^a^ Haiti, 2012–2013 ^a^ Low availability=no facility with delivery services within the buffer distance; medium availability=1 facility with delivery services within the buffer; high availability=2 or more facilities with delivery services within the buffer. The buffer distance was 5 km for urban clusters and 10 km for rural clusters.

Availability of facilities offering delivery services varied by location; there was a greater number of facilities within the buffer of clusters in the metropolitan area than in other urban areas and rural areas. The number of facilities with delivery services within the buffer distances from the DHS clusters (i.e., within 5 km for the metropolitan and other urban areas and within 10 km for rural areas) ranged from 0–28 in the metropolitan area, from 0–6 in other urban areas, and from 0–7 in rural areas. Figure 2 presents the percentage distribution of women by levels of availability to delivery services. Proximity to a facility with delivery services was nearly universal in the metropolitan area; 99% of women lived in an area with a high level of availability of delivery services (2 or more facilities within 5 km from their clusters). In contrast, 12% of women in other urban areas and 18% of rural women lived in areas with low availability—that is, no facility offering delivery services within 5 km for women in other urban areas and 10 km for women in rural areas.

Proximity to a facility with delivery services was nearly universal in the metropolitan area, whereas 12% of women in other urban areas and 18% of rural women lived in areas with no such facility.

### Facilities' Readiness to Provide Delivery Services

Readiness to provide delivery services among facilities that offered delivery services was measured by the availability of a number of basic and comprehensive obstetric care services and items available at the facility on the day of the survey. Table 1, which presents the availability of these 37 services and supplies, shows wide variation by the facility's location. Facilities in the metropolitan and other urban areas had many of the items while those in rural areas had few. Some items were commonly available across locations, such as a delivery bed, gloves, injectable antibiotics, and parenteral administration of oxytocic drug, while other items were rare, such as manual vacuum extractor and guidelines for integrated management of pregnancy and childbirth. Overall, the availability of comprehensive obstetric care items was low, especially at facilities in rural areas.

**TABLE 1.** Percentage of Facilities Offering Delivery Services With Availability of Basic and Comprehensive Obstetric Care Services and Items, by Residence, Haiti, 2013

	Rural %	Other Urban %	Metropolitan %	Total %
**Basic obstetric care indicators**				
Parenteral administration of antibiotics	68.9	78.0	94.9	77.9
Parenteral administration of oxytocic drug	86.5	95.1	94.9	91.8
Parenteral administration of anticonvulsants	40.5	67.1	48.7	53.3
Assisted vaginal delivery^a^	90.5	92.7	89.7	91.3
Manual removal of placenta	58.1	79.3	59.0	67.2
Manual removal of retained products	48.6	75.6	64.1	63.1
Neonatal resuscitation	51.4	72.0	53.8	60.5
Guidelines for IMPAC	21.6	31.7	12.8	24.1
Staff trained in IMPAC	48.6	53.7	46.2	50.3
Emergency transportaion	32.4	36.6	35.9	34.9
Sterilization equipment	75.7	84.1	87.2	81.5
Examination light	43.2	43.9	59.0	46.7
Delivery pack	87.8	92.7	87.2	89.7
Suction apparatus	93.2	98.8	79.5	92.8
Manual vacuum extractor	16.2	19.5	20.5	18.5
Vacuum aspirator or D&C kit	20.3	35.4	30.8	28.7
Newborn bag and mask	51.4	62.2	59.0	57.4
Delivery bed	97.3	100.0	94.9	97.9
Partograph	32.4	41.5	33.3	36.4
Gloves	93.2	93.9	89.7	92.8
Antibiotic eye ointment for newborns	66.2	84.1	66.7	73.8
Injectable uterotonic	62.2	74.4	64.1	67.7
Injectable antibiotics	89.2	92.7	94.9	91.8
Injectable magnesium sulphate	60.8	78.0	74.4	70.8
Skin disinfectant	60.8	81.7	61.5	69.7
Intravenous solution with infusion set	74.3	84.1	82.1	80.0
Regular reviews of maternal or newborn deaths	25.7	32.9	48.7	33.3
**Comprehensive obstetric care indicators**				
Cesarean delivery services	23.0	52.4	71.8	45.1
Blood transfusion	23.0	50.0	61.5	42.1
Guidelines for CEmOC adapted for Haiti	6.8	15.9	7.7	10.8
Staff member providing delivery trained in CEmOC	44.6	45.1	38.5	43.6
Anesthesia equipment	10.8	15.9	25.6	15.9
Incubator	10.8	23.2	23.1	18.5
Blood typing	17.6	42.7	56.4	35.9
Cross matching test	4.1	3.7	10.3	5.1
Blood supply sufficiency	12.2	19.5	20.5	16.9
Blood supply safety	16.2	37.8	53.8	32.8
**Total number of facilities**	**74**	**82**	**39**	**195**

Abbreviations: CEmOC, comprehensive emergency obstetric care; D&C, dilation and curettage; IMPAC, integrated management of pregnancy and childbirth.

a Assisted vaginal delivery was actually interpreted as "attended vaginal delivery" in the Haiti Service Provision Assessment.

Availability of comprehensive obstetric care items was low across the board, but especially in facilities in rural areas.

**FIGURE 3:**
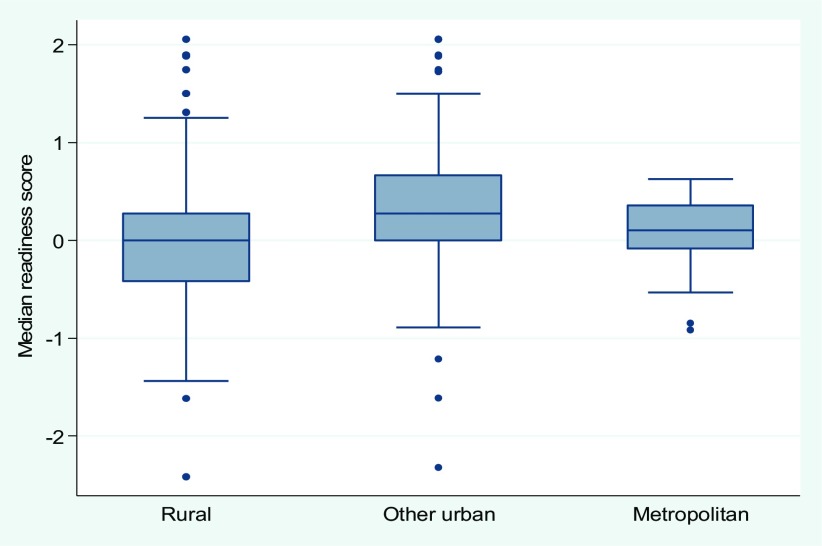
Distribution of Cluster-Level Median Readiness Scores Among Facilities Offering Delivery Services, by Residence, Haiti, 2012–2013

As discussed earlier, to assess the level of service readiness at facilities that the DHS cluster was linked to, an overall readiness score was generated for each cluster based on the median score of all facilities within the buffer. The clusters were divided into low-, medium-, and high-readiness groups based on the readiness score terciles at the cluster level. Figure 3 shows that the distribution of readiness scores differed by residence area. In the metropolitan area, the cluster readiness scores were more homogenous, while the interquartile range was wider in other urban and rural clusters. The readiness score distribution in other urban areas was highly skewed to high readiness scores, indicating that some facilities had much better readiness than others. A few outlier facilities with markedly different (better or worse) readiness compared with the rest of facilities were also observed in each residence area.

### Use of Facility Delivery Services by Level of Availability and Readiness of Delivery Services

Overall, 39% of women delivered their most recent birth at a health facility and 61% delivered at home. Delivery at a health facility was far more common in the metropolitan and other urban areas (60% and 59%, respectively) compared with rural areas (24%).

**FIGURE 4:**
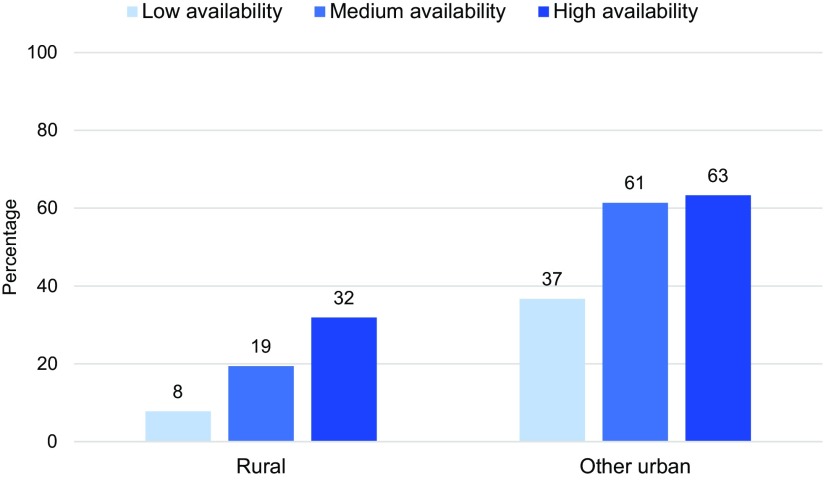
Percentage of Women Who Delivered at a Facility by Availability of Facilities With Delivery Services Within the Buffer Distance,^a^ Haiti, 2012–2013 ^a^ Low availability=no facility with delivery services within the buffer distance; medium availability=1 facility with delivery services within the buffer; high availability=2 or more facilities with delivery services within the buffer. The buffer distance was 5 km for urban clusters and 10 km for rural clusters.

Figure 4 highlights the bivariate association between availability of delivery services and the percentage of women who delivered their most recent birth at a health facility. Since nearly all women in the metropolitan area lived in clusters with 2 or more facilities within 5 km, the effect of availability of delivery services was assessed only for other urban and rural areas. In both types of area, use of facility delivery was significantly associated with levels of availability of facilities offering delivery services. In rural areas, facility delivery coverage increased incrementally with the level of availability. In rural clusters without a facility with delivery services within 10 km, only 8% of women delivered the most recent birth in a facility compared with 19% of women in clusters with access to 1 such facility, and 32% of those with 2 or more such facilities. Service availability was also associated with use in other urban areas but the difference was less remarkable between medium and high availability.

**FIGURE 5:**
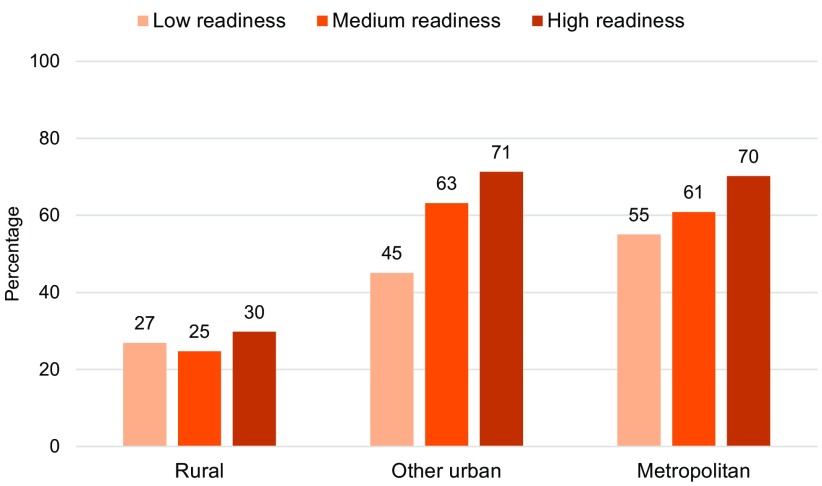
Percentage of Women Who Delivered at a Facility by Level of Readiness^a^ at Facilities With Delivery Services Within the Buffer Distance,^b^ Haiti, 2012–2013 ^a^ Low-, medium-, and high-level readiness=median readiness score of the facilities within the buffer falling in the bottom 33.3%, middle 33.3%, and top 33.3%, respectively. The buffer distance was 5 km for urban clusters and 10 km for rural clusters. ^b^ Women living in clusters with no health facility offering delivery services within the buffer distance were excluded from this figure since the readiness score was applied only to clusters with 1 or more health facilities.

Figure 5 indicates that use of facility delivery services was also positively associated with the level of readiness at the facilities within the buffer distance in the metropolitan and other urban areas. In other urban clusters, 45% of women delivered in a facility with low level of readiness compared with 63% of women for facilities with a medium level of readiness and 71% of women for facilities with a high level of readiness. In rural areas, however, there was little association between facilities' level of readiness and women's use of facility delivery services.

### Results of Multivariable Analysis

Using random-intercept logistic models, we assessed how women's use of facility delivery was associated with availability of delivery services within the buffer and service readiness of the facilities. We first ran models for nonmetropolitan urban areas and rural areas to examine the effect of availability. This model was not run for the metropolitan area since almost all metropolitan women had a high level of availability to delivery services. We then ran models to examine the effect of service readiness for all 3 locations after controlling for availability and other covariates. Since readiness is only available for clusters with at least 1 facility within the buffer distance, women in clusters with no facility were dropped from this model.

Table 2 presents odds ratios (ORs) and 95% confidence intervals (CIs) from the regression models of facility delivery on the availability indicator, as well as for the covariates adjusted for in the models. In both urban and rural areas, availability of delivery services was significantly associated with women's use of facility delivery care after controlling for other covariates. In rural areas, women in clusters with medium-level availability to delivery services had 1.9 times higher odds of going to a facility for delivery (CI, 1.14 to 3.13; *P*<.05) and women in clusters with high availability had 3.6 times higher odds (CI, 2.23 to 5.66; *P*<.001) compared with women with low availability. Similarly, in other urban areas higher availability of delivery services was also significantly associated with a greater likelihood to deliver in a facility. Although the odds ratio appears lower for high availability (OR, 2.64; 95% CI, 1.13 to 6.19; *P*<.05) than medium availability (OR, 3.83; 95% CI, 1.57 to 9.31; *P*<.01), the difference was not statistically significant (results not shown).

**TABLE 2.** Multivariable Model Results for the Regressions of Facility Delivery on Availability of Facilities With Delivery Services Within the Buffer, Controlling for Other Variables, Haiti, 2012–2013

Variables	Rural		Other Urban	
	Odds Ratio	95% CI	Odds Ratio	95% CI
**Availability of facilities with delivery services**				
Low availability	1.00		1.00	
Medium availability	1.89*	1.14, 3.13	3.83**	1.57, 9.31
High availability	3.56***	2.23, 5.66	2.64*	1.13, 6.19
**Mother's age at birth**	1.06***	1.03, 1.08	1.08***	1.04, 1.13
**Birth order**				
1	1.00		1.00	
2–3	0.30***	0.23, 0.39	0.30***	0.19, 0.48
4–5	0.15***	0.10, 0.22	0.23***	0.12, 0.46
6+	0.10***	0.06, 0.17	0.14***	0.05, 0.37
**Education**				
None	1.00		1.00	
Primary	1.37	0.99, 1.89	0.95	0.48, 1.89
Secondary or higher	1.85**	1.27, 2.68	1.64	0.80, 3.35
**Wealth quintile**				
Lowest	1.00		NA^a^	NA^a^
Second	1.86***	1.38, 2.50	NA^a^	NA^a^
Middle	2.99***	2.12, 4.20	1.00	
Fourth	5.72***	3.61, 9.07	1.43	0.91, 2.25
Highest	10.73***	4.83, 23.80	3.39***	1.91, 6.04
**Antenatal care visits**				
None	1.00		1.00	
1	4.46***	2.05, 9.69	1.38	0.36, 5.29
2–3	3.90***	2.09, 7.28	1.40	0.51, 3.82
4+	6.75***	3.71, 12.27	2.85*	1.13, 7.17
**Departement**				
Ouest	1.00		1.00	
Sud-est	1.17	0.61, 2.23	2.96	0.73, 12.08
Nord	0.92	0.53, 1.60	1.83	0.64, 5.28
Nord-est	1.62	0.79, 3.32	1.74	0.53, 5.78
Artibonite	1.17	0.76, 1.78	1.37	0.49, 3.86
Centre	1.64	0.97, 2.78	1.42	0.39, 5.20
Sud	1.56	0.94, 2.59	1.90	0.52, 6.88
Grand'anse	1.21	0.57, 2.57	0.68	0.17, 2.73
Nord-ouest	1.04	0.56, 1.93	0.67	0.19, 2.39
Nippes	1.35	0.67, 2.70	1.71	0.26, 11.14
**Number of clusters**	**241**		**85**	
**Observations**	**2,878**		**829**	

a In other urban areas, no women were in the first wealth quintile and very few women were in the second wealth quintile; they were combined into the third quintile.

***P<.001; **P<.01; *P<.05.

In both urban and rural areas, availability of delivery services was significantly associated with women's use of facility delivery care.

As expected, maternal age at birth, the number of antenatal care visits, and household wealth were positively and significantly associated with women's use of delivery care in a health facility, while the child's birth order was negatively associated.

Table 3 shows the results of the regression models of facility delivery on service readiness and availability after controlling for women's characteristics and antenatal care variables. Service readiness was significantly associated with facility delivery only in urban areas (other than the metropolitan area). Women in clusters with a high level of service readiness had 2.7 times higher odds of delivery in a health facility compared with women in clusters with low readiness (95% CI, 1.34 to 5.60; *P*<.01). The difference in facility delivery between women in clusters with low readiness and those with medium readiness was not significant after controlling for other covariates. Similar to the findings from the models on availability, in other urban areas there was no significant difference in coverage of facility delivery between clusters with medium availability and clusters with high availability of delivery services. In rural areas, after controlling for service readiness and other variables, having high availability of delivery services—that is, having 2 or more facilities within the buffer distance—was associated with significantly greater odds of delivery in a health facility compared with medium availability—that is, having only 1 facility with delivery services within the buffer distance (OR, 1.94; 95% CI, 1.38 to 2.72; *P*<.001).

**TABLE 3.** Multivariable Model Results for the Regressions of Facility Delivery on Service Readiness of Facilities With Delivery Services, Controlling for Availability of Services and Other Variables, Haiti, 2012–2013

Variables	Rural		Other Urban		Metropolitan	
	Odds Ratio	95% CI	Odds Ratio	95% CI	Odds Ratio	95% CI
**Facilities' readiness to provide delivery services**^a^						
Low readiness	1.00		1.00		1.00	
Medium readiness	1.43	0.93, 2.18	1.74	0.71, 4.26	1.02	0.74, 1.41
High readiness	1.33	0.95, 1.85	2.74**	1.34, 5.60	1.41	0.96, 2.05
**Availability of facilities with delivery services**^b^						
Medium availability	1.00		1.00		NA	
High availability	1.94***	1.38, 2.72	0.72	0.37, 1.41	NA	
**Mother's age at birth**	1.05***	1.03, 1.08	1.09***	1.05, 1.14	1.023	0.99, 1.05
**Birth order**						
1	1.00		1.00		1.00	
2–3	0.31***	0.23, 0.42	0.28***	0.17, 0.47	0.61**	0.43, 0.88
4–5	0.15***	0.10, 0.24	0.22***	0.10, 0.48	0.50*	0.29, 0.86
6+	0.12***	0.07, 0.20	0.17**	0.06, 0.49	0.42*	0.20, 0.91
**Education**						
None	1.00		1.00		1.00	
Primary	1.47*	1.04, 2.08	0.91	0.42, 1.96	3.23***	1.71, 6.12
Secondary or higher	2.06***	1.39, 3.06	1.91	0.86, 4.24	5.01***	2.63, 9.55
**Wealth quintile**^c^						
Lowest	1.00		NA^c^	NA^c^	NA^c^	NA^c^
Second	1.89***	1.37, 2.61	NA^c^	NA^c^	NA^c^	NA^c^
Middle	3.04***	2.11, 4.38	1.00		1.00	
Fourth	5.72***	3.56, 9.21	1.47	0.87, 2.46	1.05	0.68, 1.63
Highest	11.14***	4.95, 25.07	2.82**	1.49, 5.35	2.56***	1.55, 4.20
**Antenatal care visits**						
None	1.00		1.00		1.00	
1	4.75***	2.05, 10.99	2.02	0.47, 8.73	3.60*	1.25, 10.32
2–3	4.17***	2.13, 8.17	2.16	0.69, 6.76	2.20*	1.12, 4.32
4+	7.24***	3.81, 13.79	3.88*	1.36, 11.05	3.95***	2.17, 7.18
**Departement**						
Ouest	1.00		1.00			
Sud-est	0.98	0.47, 2.03	1.00	0.11, 9.03		
Nord	0.89	0.50, 1.57	2.00	0.57, 6.98		
Nord-est	1.63	0.79, 3.39	2.37	0.55, 10.15		
Artibonite	1.19	0.75, 1.88	1.60	0.46, 5.62		
Centre	1.64	0.92, 2.92	1.92	0.46, 7.94		
Sud	1.62	0.95, 2.76	2.46	0.48, 12.64		
Grand'anse	0.97	0.40, 2.34	0.58	0.12, 2.79		
Nord-ouest	1.02	0.54, 1.92	0.65	0.16, 2.68		
Nippes	1.36	0.66, 2.81	2.67	0.31, 22.83		
**Number of clusters**	**201**		**69**		**59**	
**Observations**	**2,571**		**802**		**650**	

a Regressions excluded women living in clusters with no health facility offering delivery services within the buffer since the readiness score is applied only to clusters with 1 or more health facilities.

b Since the analysis excluded women living in clusters with no health facility offering delivery services within the buffer (i.e., the low availability group), the medium availability group was set as the reference.

c In the metropolitan and other urban areas, no women were in the first wealth quintile and very few women were in the second wealth quintile; they were combined into the third quintile.

***P<.001; **P<.01; *P<.05.

Service readiness was significantly associated with facility delivery only in urban areas.

## DISCUSSION

The availability of the recent DHS and SPA surveys in Haiti, both with GPS data, enabled analysis linking women's use of facility delivery care with their physical access to delivery care and facilities' readiness to provide the care. This analysis showed that women in rural areas of Haiti have very limited physical access to obstetric care. Almost 1 in 5 women in rural areas lives in a place where there is no facility that provides delivery services within a 10 km distance. It should be noted that the 10 km distance is a straight-line measurement. The actual travel distance could be longer because of travel paths, road networks, and the mountainous terrain. In rural areas, physical access to health care can be further constrained by poor road conditions and lack of transportation.

Women in rural areas of Haiti have very limited physical access to obstetric care.

The regression results highlight the importance of service availability to the use of facility delivery in rural areas. Living reasonably near a facility that provides delivery services is significantly associated with greater probability of women delivering at a health facility. Physical access as an important barrier to service use in rural Haiti was also demonstrated in previous research that measured access with the distance to the nearest facility reported by key community informants.^23^ Our finding also agrees with previous research that having physical access to services is a strong determinant of use of delivery care.^28^^,^^34^^–^^36^

Living reasonably near a facility that provides delivery services is significantly associated with greater probability of women delivering at a health facility.

Quality of care—measured in our study by analyzing how prepared the facility is to provide the needed services—is important to the use of health services. Our results show that hospitals and health centers with a bed are not well prepared to provide delivery services. A large number of facilities lack essential equipment and supplies for routine delivery care, and most have limited capacity to provide emergency obstetric care. Only about one-third of facilities have functional emergency transportation, and the availability of Cesarean delivery services is limited, especially in rural areas. Delivery in a health facility itself cannot reduce maternal mortality unless women are assisted by a skilled birth attendant capable of managing common life-threatening obstetric complications.^38^ In Haiti, however, less than half of the facilities have staff who received training in comprehensive emergency obstetric care (CEmOC) during the last 2 years. Guidelines for CEmOC were not available in service areas at most facilities. All of these limitations increase the risk of death for mother and newborn when an obstetrical emergency occurs.

Hospitals and health centers with a bed in Haiti are not well prepared to provide delivery services.

Despite the poor readiness of health facilities to provide delivery services, service quality appears to play an important role predicting facility delivery in urban areas, where delivery services are more available, compared with rural areas. Urban areas have more facilities, more accessible transportation, and more financial resources, and thus women may be able to choose to deliver at facilities offering a better quality of care. In rural areas, however, especially in mountainous areas, there is less availability of facilities and thus having access to at least 1 health facility seems more important to the use of delivery care than the facility's level of readiness to provide the services.

We did not find an association between service readiness and use of facility delivery in the metropolitan area. This could be because a 5 km buffer may not be appropriate for defining the service environment for clusters in the metropolitan area. Because of a high density of health facilities and DHS clusters in the metropolitan area, using a 5 km buffer may result in adjacent clusters linking to more or less the same group of facilities; therefore, there is limited variation across metropolitan clusters in terms of the service environment. This is indicated by less variation in readiness among clusters in the metropolitan area compared with clusters in rural and other urban areas. Moreover, people in metropolitan areas are likely to have more transportation options available and better accessibility to health services that are geographically further from their home.

### Strengths and Limitations

As discussed, because of methodological constraints in linking population data and health service data, most previous studies have been limited to measuring service provision from the client's perspective. Several recent studies have taken advantage of geographic data to associate health facilities and DHS clusters. These studies focused primarily on distance to the closest facility or the service available in the closest facility; however, this approach can be problematic because DHS cluster locations have been displaced. Our study improved on this methodology; instead of looking at a single facility (the closest facility)—where estimates may be subject to misclassification errors—we measured the effect of the service environment within a reasonable distance. In addition to the methodological improvement of measuring the service environment, some of the other strengths of our study lie in the use of facility census data and nationally representative household data, measuring service readiness with a wide range of items that the World Health Organization has identified as essential for providing high-quality delivery services. Together, these improvements in methodology have led to more generalizable results. Additionally, the use of observed availability of equipment and items instead of self-reported data during facility data collection increases the robustness of the readiness indicators and thus the accuracy of the relationship between the provision of delivery services and their use.

Linking women's use of health services to the service environment within a reasonable distance from DHS clusters is less prone to misclassification errors (resulting from the displacement of DHS clusters) than linking to the closet facility.

This study also has some limitations. One limitation is the temporal gap between the outcome variables and the service variables. Facility data reflect the "current" service environment at the time of the Haiti 2013 SPA, while use of facility delivery was measured over a 5-year time period preceding the 2012 Haiti DHS. Associating service provision and facility delivery use data could be problematic if the service environment changed substantially over this time period. Given the nature of delivery services, however, we do not expect that there was a substantial change in the availability of services and facilities' readiness to provide the service over the period.

While linking women to all of the facilities that they likely used reduces misclassification errors from the DHS GPS displacement procedure, the straight-line buffer approach does not take into account the mountainous terrain or the impassibility of roads during the rainy season, which may limit access to a linked facility. However, Nesbitt and colleagues compared 6 different measures of spatial access and found that the straight-line linkage yields results similar to other geospatial algorithms in a developing-country setting.^20^ Finally, the buffer linkage between DHS clusters and SPA facilities may not be appropriate in areas where there is a high density of both health facilities and population, such as Haiti's metropolitan area. More precise measurements of the service environment are needed for such areas, as well as a better understanding of other drivers of use in areas where service availability may be less of an issue.

## CONCLUSION

This study indicates the importance of improving physical access to delivery services in rural Haiti. Overall, health facilities in Haiti are poorly equipped and do not appear ready to provide high-quality delivery services. Improving the quality of care at health facilities could contribute to increased use of facility delivery particularly in nonmetropolitan urban areas, where 40% of women still deliver at home. Over the years, the global community has recognized the importance of providing women with quality maternal health services to reduce maternal mortality.^38^ After all, reducing maternal mortality by having women deliver in health facilities will only work if these facilities are ready to provide comprehensive obstetric care.

## Supplementary Material

Supplementary Table 1

## Notes

**Peer Reviewed**
