# Supplementary material for: Limited Service Availability, Readiness, and Use of Facility-Based Delivery Care in Haiti: A Study Linking Health Facility Data and Population Data
Source: Glob Health Sci Pract. 2017 Jun 27;5(2):244–60. doi: 10.9745/GHSP-D-16-00311 (PMC5487087; doi:10.9745/GHSP-D-16-00311)
Supplement: Supplementary Table 1 [file GHSP-D-16-00311_index.html]

Supplement to Limited Service Availability, Readiness, and Use of Facility-Based Delivery Care in Haiti: A Study Linking Health Facility Data and Population Data | Global Health: Science and Practice

## Supplements

SUPPLEMENTARY TABLE 1. Operational Definitions of Service Readiness Indicators

SUPPLEMENTARY TABLE 2. Percentage Distribution of Health Facilities That Provide Normal Delivery Services by Facility Background

SUPPLEMENTARY TABLE 3. Characteristics of Women Who Have Had a Live Birth in the Five Years Preceding the Survey, Haiti DHS 2012

**Files in this Data Supplement:**

- Supplement 1 - Text s01, PDF
- Supplement 2 - Text s02, PDF
- Supplement 3 - Text s03, PDF
