# Supplementary material for: Limited Service Availability, Readiness, and Use of Facility-Based Delivery Care in Haiti: A Study Linking Health Facility Data and Population Data
Source: Glob Health Sci Pract. 2017 Jun 27;5(2):244–60. doi: 10.9745/GHSP-D-16-00311 (PMC5487087; doi:10.9745/GHSP-D-16-00311)
Supplement: Supplementary Table 1 [file 16-00311-Wang-Supplementary-Table1.pdf]

Wang W, Winner M, Burgert-Brucker CR. Limited service availability, readiness, and use of facility-based delivery care in Haiti: a study linking health facility data and population data. *Glob Health Sci Pract*. 2017;5(2). https://doi.org/10.9745/GHSP-D-16-00311

SUPPLEMENTARY TABLE 1. Operational Definitions of Service Readiness Indicators

**Readiness Indicator**	**Operational Definition**
**Basic obstetric care**	
Parenteral administration of antibiotics	Respondent reports that intervention has been carried out at least once during the past 3 months by providers as part of their work in that facility
Parenteral administration of oxytocic drug	Respondent reports that intervention has been carried out at least once during the past 3 months by providers as part of their work in that facility
Parenteral administration of anticonvulsants	Respondent reports that intervention has been carried out at least once during the past 3 months by providers as part of their work in that facility
Assisted vaginal delivery[a]	Respondent reports that intervention has been carried out at least once during the past 3 months by providers as part of their work in that facility
Manual removal of placenta	Respondent reports that intervention has been carried out at least once during the past 3 months by providers as part of their work in that facility
Manual removal of retained products	Respondent reports that intervention has been carried out at least once during the past 3 months by providers as part of their work in that facility
Neonatal resuscitation	Respondent reports that intervention has been carried out at least once during the past 3 months by providers as part of their work in that facility
Guidelines for IMPAC	Guidelines for Integrated Management of Pregnancy and Childbirth observed by interviewer in the service site
Staff trained in IMPAC	Respondent reports having received in-service training or training updates in Integrated Management of Pregnancy and Childbirth in the past 24 months
Emergency transportation	Respondent reports that the facility has a functional ambulance or other vehicle for emergency transportation for clients stationed at and operating from that facility or has access to one stationed at or operating from another facility and fuel is available for that vehicle on the day of interview
Sterilization equipment	Sterilization equipment observed by interviewer anywhere in the facility and reported functioning by respondent. Equipment may be electric (autoclave, dry heat sterilizer, boiler or steamer) or non-electric (autoclave or pot for boiling). If non-electric, facility must have a stove or cooker as a heat source.
Examination light	Functioning spotlight source that can be used for patient examination observed by interviewer in delivery area and reported functioning by respondent
Delivery pack	Delivery pack (or all of the following: cord clamp, episiotomy scissors, scissors or blade to cut cord, suture material and needle holder) observed by interviewer in delivery area

Wang W, Winner M, Burgert-Brucker CR. Limited service availability, readiness, and use of facility-based delivery care in Haiti: a study linking health facility data and population data. *Glob Health Sci Pract*. 2017;5(2). https://doi.org/10.9745/GHSP-D-16-00311

**Readiness Indicator**	**Operational Definition**
Suction apparatus (mucus extractor)	Suction bulb or suction apparatus with catheter observed by interviewer in delivery area and reported functioning by respondent
Manual vacuum extractor	Manual vacuum extractor observed by interviewer in delivery area and reported functioning by respondent
Vacuum aspirator or D&C kit	Vacuum aspirator or D&C kit observed by interviewer in delivery area and reported functioning by respondent
Newborn bag and mask	Newborn bag and mask observed by interviewer in delivery area and reported functioning by respondent
Delivery bed	Delivery bed observed by interviewer in delivery area
Partograph	Blank partographs observed by interviewer in delivery area
Gloves	Disposable latex gloves observed by interviewer in delivery area
Antibiotic eye ointment for newborns	Tetracycline or other antibiotic eye ointment for newborn observed by interviewer in delivery area or in the main location in the facility where medicines and commodities are routinely stored
Injectable uterotonic	Oxytocin or other injectable uterotonic observed by interviewer in delivery area or in the main location in the facility where medicines and commodities are routinely stored
Injectable antibiotics	Injectable antibiotic (gentamycin, Benzathine Benzylpenicillin, Ceftriaxone, ampicillin or metronidazole injection) observed by interviewer in delivery area or in the main location in the facility where medicines and commodities are routinely stored
Injectable magnesium sulphate	Injectable magnesium sulphate observed by interviewer in delivery area or in the main location in the facility where medicines and commodities are routinely stored
Skin disinfectant	Skin disinfectant observed by interviewer in delivery area or in the main location in the facility where medicines and commodities are routinely stored
Intravenous solution	Intravenous solution (plasma expanders) with infusion set (Normal saline or Ringers Lactate, or Dextrose 5%) observed by interviewer in delivery area or in the main location in the facility where medicines and commodities are routinely stored
Regular reviews of maternal or newborn deaths	Respondent reports that facility participates in regular reviews of maternal or newborn deaths of 'near misses'

Wang W, Winner M, Burgert-Brucker CR. Limited service availability, readiness, and use of facility-based delivery care in Haiti: a study linking health facility data and population data. *Glob Health Sci Pract*. 2017;5(2). https://doi.org/10.9745/GHSP-D-16-00311

**Readiness Indicator**	**Operational Definition**
**Comprehensive obstetric care**	
Cesarean section services	Respondent reports that the facility offers cesarean section
Blood transfusion	Respondent reports that the facility offers blood transfusion services
Guidelines for CEmOC adapted for Haiti	Guidelines for comprehensive emergency obstetric care observed by interviewer in the delivery area
Staff member providing delivery trained in CEmOC	Respondent reports having received in-service training or training updates in CemOC in the past 24 months
Anesthesia equipment	Anesthesia equipment (all of the following: anesthesia machine, tubings and connectors to connect to the endotracheal tube, stylet, pediatric and adult resuscitator bag and mask, oropharyangeal airway, endotracheal tubes, Magill's forceps) observed by interviewer in the delivery area and reported functioning by respondent
Incubator	Incubator observed by interviewer in delivery area and reported functioning by respondent
Blood typing	Blood group tests (Anti-A, Anti-B and Anti-D reagents) and centrifuge for CSF microscopy observed by interviewer in facility and reported functioning by respondent
Cross matching test	Blood group tests (Anti-A, Anti-B and Anti-D reagents), centrifuge for CSF microscopy, Coomb's reagent and incubator observed by interviewer in facility and reported functioning by respondent
Blood supply sufficiency	Respondent reports that the facility offers blood transfusion services and has not run out of blood for more than one day at any time during the past 3 months
Blood supply safety	Respondent reports that the facility offers blood transfusion services and either obtains blood only from national or regional blood banks or obtains blood from other sources but screened for HIV, syphilis, hepatitis B and hepatitis C

Abbreviations: CSF, cerebrospinal fluid; CEmOC, comprehensive emergency obstetric care; SARA, Service Availability and Readiness Assessment (from the World Health Organization); SPA, Service Provision Assessment.

Note: A few indicators were defined somewhat differently from the definitions shown in the SARA manual; this was done so they could be constructed using the available SPA data. For example, the SARA manual includes a laryngoscope among the list of equipment necessary for administering anesthesia;

Wang W, Winner M, Burgert-Brucker CR. Limited service availability, readiness, and use of facility-based delivery care in Haiti: a study linking health facility data and population data. *Glob Health Sci Pract*. 2017;5(2). https://doi.org/10.9745/GHSP-D-16-00311

however, the Haiti SPA questionnaire did not ask about the availability of laryngoscopes. In addition to the SARA indicators, “regular reviews of maternal or newborn deaths” was added to the list of readiness indicators.

[a] Assisted vaginal delivery was actually interpreted as "attended vaginal delivery" in the Haiti SPA.
