# Supplementary material for: Limited Service Availability, Readiness, and Use of Facility-Based Delivery Care in Haiti: A Study Linking Health Facility Data and Population Data
Source: Glob Health Sci Pract. 2017 Jun 27;5(2):244–60. doi: 10.9745/GHSP-D-16-00311 (PMC5487087; doi:10.9745/GHSP-D-16-00311)
Supplement: Supplementary Table 1 [file 16-00311-Wang-Supplementary-Table2.pdf]

Wang W, Winner M, Burgert-Brucker CR. Limited service availability, readiness, and use of facility-based delivery care in Haiti: a study linking health facility data and population data. *Glob Health Sci Pract*. 2017;5(2). https://doi.org/10.9745/GHSP-D-16-00311

SUPPLEMENTARY TABLE 2. Percentage Distribution of Health Facilities That Provide Normal Delivery Services by Facility Background Characteristics, Haiti SPA 2013

Facility Characteristics	Rural<br>%	Other Urban<br>%	Metropolitan<br>%	Total<br>%
**Type of facility**				
Hospital	24.3	57.3	74.4	48.2
Health center with bed	75.7	42.7	25.6	51.8
**Managing authority**				
Government	35.1	62.2	17.9	43.1
NGO/private not-for-profit	16.2	7.3	23.1	13.8
Private for-profit	25.7	12.2	56.4	26.2
Mixed[a]	23.0	18.3	2.6	16.9
**Departement**				
Ouest	28.4	13.4	100.0	36.4
Sud-Est	2.7	6.1	0.0	3.6
Nord	10.8	15.9	0.0	10.8
Nord-Est	8.1	4.9	0.0	5.1
Artibonite	20.3	12.2	0.0	12.8
Centre	10.8	9.8	0.0	8.2
Sud	4.1	13.4	0.0	7.2
Grand-Anse	2.7	7.3	0.0	4.1
Nord-Ouest	8.1	9.8	0.0	7.2
Nippes	4.1	7.3	0.0	4.6
**Total number of facilities**	**74**	**82**	**39**	**195**

Abbreviation: SPA, Service Provision Assessment.

[a] Mixed facilities are those private nonprofit facilities that also receive subsidies or salaried regular staff from the government.
