# Supplementary material for: Limited Service Availability, Readiness, and Use of Facility-Based Delivery Care in Haiti: A Study Linking Health Facility Data and Population Data
Source: Glob Health Sci Pract. 2017 Jun 27;5(2):244–60. doi: 10.9745/GHSP-D-16-00311 (PMC5487087; doi:10.9745/GHSP-D-16-00311)
Supplement: Supplementary Table 1 [file 16-00311-Wang-Supplementary-Table3.pdf]

Wang W, Winner M, Burgert-Brucker CR. Limited service availability, readiness, and use of facility-based delivery care in Haiti: a study linking health facility data and population data. *Glob Health Sci Pract*. 2017;5(2). https://doi.org/10.9745/GHSP-D-16-00311

SUPPLEMENTARY TABLE 3. Characteristics of Women Who Have Had a Live Birth in the Five Years Preceding the Survey, Haiti DHS 2012

	Rural		Other Urban		Metropolitan	
**Background Characteristics**	**%**	**N**	**%**	**N**	**%**	**N**
**Maternal age at birth**						
<20	13.7	396	13.8	115	15.3	186
20–34	65.3	1,879	71.9	596	69.4	843
35–49	21.0	604	14.3	118	15.2	185
**Birth order**						
1	26.8	773	39.6	328	42.3	514
2–3	35.1	1,010	37.9	314	37.7	458
4–5	18.4	530	14.3	119	13.6	166
6+	19.7	566	8.1	67	6.4	78
**Maternal education**						
None	26.8	772	8.8	73	8.5	103
Primary	48.1	1,384	34.5	286	32.1	390
Secondary or higher	25.1	722	56.7	470	59.4	721
**Wealth quintile**						
Lowest	36.4	1,046	0.0	0	0.0	0
Second	34.4	991	0.7	6	0.3	4
Middle	19.7	567	25.7	213	13.9	168
Fourth	7.7	222	40.2	333	43.2	524
Highest	1.8	52	33.4	277	42.6	517
**Number of antenatal care visits**						
None	11.7	336	4.8	40	6.8	83
1	4.6	133	3.0	25	2.2	26
2–3	23.7	681	12.3	102	13.5	164
4+	60.0	1,726	78.4	650	75.9	921
Don't know/missing	0.1	3	1.4	12	1.6	19
**Total**	**100.0**	**2,878**	**100.0**	**829**	**100.0**	**1,214**

Abbreviation: DHS, Demographic and Health Survey.
